# Challenges experienced by newly qualified nurse-midwives transitioning to practice in selected midwifery settings in northern Malawi

**DOI:** 10.1186/s12912-022-01012-y

**Published:** 2022-08-25

**Authors:** Mathews Brave Mtegha, Elizabeth Chodzaza, Ellen Chirwa, Fatch Welcome Kalembo, Maggie Zgambo

**Affiliations:** 1 Department of Nursing and Midwifery, St Johns Institute for Health, Mzuzu, Malawi; 2 School of Maternal, Neonatal and Reproductive Health, Kamuzu University of Health Sciences, Blantyre, Malawi; 3 School of Nursing, Curtin University, Perth, Australia; 4 School of Nursing and Midwifery, Edith Cowan University, Perth, Australia

**Keywords:** Newly qualified midwives, Transition experience, Transition support programs, Transition challenges of midwives, Transition to practice

## Abstract

**Background:**

Literature shows that newly qualified nurse-midwives face challenges integrating into the workforce during their transition period from education to practice. However, little is known about the needs and challenges of Malawian nurse-midwives during their transition from education to practice. The aim of the study was to explore the transition experiences of newly qualified nurse-midwives working in selected midwifery units in Northern Malawi.

**Methodology:**

A qualitative descriptive approach was used. Data were collected through in-depth interviews using semi-structured interview guides from a purposive sample of 19 participants (13 newly qualified nurse-midwives and 6 key informants). The researchers developed two interview guides; one for the newly qualified nurse-midwives and another one for the key informants. The interview guides had questions related to newly qualified nurse-midwives experiences of transitioning to practice and the support they received. Participants were from three selected hospitals in the Northern part of Malawi that have maternity units. Data were analysed manually using thematic analysis.

**Findings:**

Five themes related to challenges faced by newly qualified nurse-midwives during their transition to practice in midwifery units emerged from the thematic analysis of the data. These included (1) Theory—practice gap, (2) Lack of confidence and skills, (3) Inadequate resources, (4) Transition support system, and (5) Workplace conflict.

**Conclusion:**

Newly qualified nurse-midwives in Malawi encounter many challenges while transitioning from education to practice. The study findings underscore the need to develop a national framework support system that could not only help newly qualified midwives adjust positively to their new role but also create more opportunities for learning and developing and strengthening a collaborative partnership between colleges and hospitals.

**Supplementary Information:**

The online version contains supplementary material available at 10.1186/s12912-022-01012-y.

## Introduction

The transition into an initial role as a practising midwife can be exciting for some newly qualified nurse-midwives and overwhelming for others [1, 2]. Evidence reveals that the transition to practice for many newly qualified nurse-midwives is characterised by learning and adapting to a new role, a change of identity, taking on new responsibilities and independence to practice [3, 4]. In most cases, the transition period usually lasts from 12 to 18 months, with the first six months being used for orientation and adjusting to the new environment [5, 6]. During this period, experienced nurse-midwives are critical in imparting nursing and midwifery skills and knowledge to newly qualified nurse-midwives until they become competent. While the transition period is important for building competence and confidence in practising nurse-midwives, authors of studies conducted in Australia, Canada, New Zealand and the United Kingdom have reported the challenges encountered by newly qualified nurse-midwives that interfere with the smooth transition into their new roles [2, 3, 5, 7]. These challenges are not limited to conflicts within the workplace, lack of experienced mentors, bullying, reality shock, high workloads, performance anxieties and poor job satisfaction [3, 5, 8, 9], and have often resulted in high turnover rates around the globe [7].

In an effort to retain nurses and midwives through professional development, many countries in both developed and undeveloped regions have structured support programs to aid a successful transition to practice [1, 3, 5, 10]. In these formal support programs, they are some commonalities on how the new graduates are supported, mentored and supervised by experienced midwives for a period to gain autonomy, competency and confidence in their new roles [3, 10, 11]. However, different models are used, for example, in New Zealand, a fully government-funded programme called the Midwifery First Year of Practice (MFYP) is used to support new graduate midwives [12]. Similarly, new graduate midwives in Australia are employed by public and private hospitals under a transition to practice programme, which offers mentorship support, educational seminars and experience in different settings within the maternity services through clinical rotations [5]. In the same country, a small group of midwives, including graduates, work together in midwifery continuity of care models, where mentors assist new graduate midwives to build confidence and practice skills [8]. In the United Kingdom, the preceptorship model is used to provide new graduate nurses with professional development [13] and South Africa which is one of the sub-Saharan African countries they have community service program [11]. On the other hand, such formal transitioning programmes do not exist in most developing countries including Malawi.

Most of these newly graduated nurse-midwives in Malawi are recruited in public hospitals where formal programs to support newly qualified nurse-midwives are not available. The importance of transition programs for newly qualified health workers is well documented and it includes supporting newly qualified midwives to strengthen their clinical competencies [14–16] and reducing attrition of staff [7, 10]. According to Malawi’s Nurses and Midwives Act no.16 of 1995, after obtaining nursing and midwifery qualifications and passing the registration examination delivered by the Nurses and Midwives Council of Malawi, newly qualified nurse-midwives are deemed independent to practice. The majority of newly qualified nurse-midwives are employed into public and private hospitals without transition support programs. However, findings from recent studies suggest that newly qualified nurse-midwives experience a successful transition to practice if supported by transition support programs as they depend on assistance from experienced colleagues [3, 10, 17]. This is further augmented by many authors who claim that newly qualified nurse-midwives do not perform to the expected standards due to a lack of confidence and competence [3, 9, 18]. Therefore, long-term and ongoing career development is essential to improve newly qualified nurse-midwives performance, which is relevant to the provision of quality midwifery care and retention of midwives in the industry.

According to a recent report by the World Health Organization, Malawi has a density of 0.33 nurses per 1000 population and the number of nurses and midwives declined by 17% between 2005 and 2018 [19]. This decline is alarming considering that the density of doctors, nurses and midwives is correlated strongly with maternal, child and infant mortality [19]. Malawi has a high estimated maternal mortality rate of 439 per 100,000 live births [20]. According to the 2019 database, Nurses and Midwives Council of Malawi (NMCM), approximately an average of 340 registered nurse-midwives and 690 nurse-midwife technicians graduate every year in Malawi from nationally accredited educational institutions [21]. In Malawi, a registered nurse-midwife is an NMCM endorsed professional who has successfully completed a bachelor’s degree program in midwifery plus any other qualification in nursing; has a bachelor’s degree in nursing and another lower qualification, such as a diploma or certificate in midwifery; or has an integrated university (or equivalent) diploma in nursing and midwifery. A qualified nurse-midwife technician has an integrated college nursing and midwifery diploma [22]. The need for innovative strategies to retain nurses and midwifery in the health system is therefore not overstated. To date, research on the needs and transitioning experiences of new graduate midwives into practice in Malawi is lacking despite evidence supporting that positive transition experiences could promote positive professional development and increase the retention of nurses and midwives in the healthcare workforce [23]. Authors of a recent study in Malawi have reported that experienced midwives and stakeholders lament a lack of confidence, skills and competencies of the newly qualified nurse-midwives [24], which could be contributing to the lowering of standards of nursing and midwifery care [25]. It is essential, therefore, to explore and understand the needs, experiences and support system for newly qualified nurse-midwives in Malawi as they transition from a structured educational environment to independent practising nurse-midwives. This evidence has the potential to inform policies and strategies to effectively support newly qualified nurse-midwives during the transition period.

## Methodology

### Study design

This study utilised a qualitative descriptive approach**.** Qualitative research helps a researcher to have a rich understanding of a phenomenon as it exists in a natural rather than experimental setting [26]. The descriptive approach involves a systematic, interactive and in-depth approach that yields subjective and rich data used to describe human experiences [26, 27]. The approach was ideal in this study because it offers a comprehensive summary of the phenomena where there is a dearth of knowledge within a particular subject, and it also addresses the ‘who, what and where’ of participants’ experiences [28]. This approach was deemed relevant for this study [8, 28].

### Study setting

The study was conducted at three hospitals selected based on the type of maternity services provided and these are Mzuzu Central Hospital, Mzimba South District Hospital and St John’s Hospital all in the northern region of Malawi. These hospitals have maternity units that provide antenatal, labour and birth, and postnatal services. Mzuzu Central Hospital and Mzimba South District Hospital are public hospitals with cost-free services, while St John’s Hospital is run by the Christian Health Association of Malawi (CHAM) and clients pay for the services. Mzuzu Central Hospital is a referral facility for all hospitals in the northern region. Overall, the maternity unit (with antenatal ward, labour and birth ward, and postnatal ward) at Mzuzu Central Hospital has 54 nurse-midwives against an average of 402 deliveries a month. There are 22 nurse-midwives against an average of 400 monthly deliveries at at Mzimba South District Hospital. St Johns Hospital has 12 nurse midwives allocated to the maternity unit with an average of 160 deliveries in a month.

### Selection criteria

The study included newly qualified and practising nurse-midwives and key informants from these hospitals. The newly qualified nurse-midwives were recruited in the study if they had completed undergraduate nursing and midwifery education in Malawi (diploma and degree) and if they had less than two years of transitioning period in practice. Key informants were recruited if they were nursing and midwifery managers (matrons, district nursing officers or chief nursing officers) working in maternity units. The researchers targeted key informants who had been in management positions for more than a year as they were considered experienced.

### Study population

This study used the purposive sampling technique to recruit participants for in-depth interviews. Purposive sampling helps to recruit participants who are knowledgeable about the phenomena under study [26, 29]. MM verbally invited fifteen potential participants assigned to maternity units in these hospitals. The participants were briefed on the study aims and procedures and voluntarily accepted to participate in the study. During the briefing, the researcher allowed the participants to ask questions for clarification and opportunity was granted to decline or withdraw participation at any time during the study. Thirteen expressed their interest to participate in the study and were further provided with written study information to provide written informed consent for autonomy [30]. We enrolled 13 newly qualified nurse-midwives for interviews, but data saturation was reached at the tenth interview. However, data collection continued for the next three participants to enhance the credibility of the findings. To enrich the data, six key informants were approached in their respective offices, and briefed about the study objectives and benefits of participating in the study. They all assented to participate and were recruited for the study.

### Data collection procedure

MM, who is a lecturer at a nursing school and had no prior relationship with participants, undertook all in-depth interviews from 4^th^ July 2019 to 23^rd^ September 2019. The interviews for new graduates were conducted in a quiet room inside the maternity unit. Key informants were interviewed in their respective offices. Two different interview guides were developed, one for the new graduates and another one for the key informants (see Additional file 1). The study objectives and literature review guided the development of the interview guides and all interviews lasted between 20 and 35 min and were digitally recorded**.** Participants voluntary consented to record their voices during the recruitment process and this was confirmed prior to interviews. Field notes were taken soon after each interview.

### Ethical considerations

The research was approved by the University of Malawi College of Medicine Research and Ethics Committee reference number P.04/19/2647. Further ethical approval was obtained from the research ethics committees of the three hospitals. Before participation, verbal and written informed consent was sought from the participants. To ensure anonymity, participants were assigned codes and names were not used in the study. After data analysis, the transcripts were kept according to the university’s data management procedures.

### Data analysis

The raw data from in-depth interviews were transcribed and analyzed manually. Each recorded interview was transcribed verbatim by MM, who also verified and corrected errors in the transcription by re-reading the transcribed data while listening to the recorded data. Data analysis followed the six phases of thematic data analysis proposed by Braun & Clarke, (2006) which are: familiarizing with data, generating initial codes, searching for themes, reviewing themes, defining and naming themes and producing the report [31].

Firstly, transcribed data from in-depth interviews were read through multiple times by the first author to familiarise himself with the data and the preliminary ideas of analytical interest that helped to organize the data were recorded. Thereafter, a line-by-line analysis of transcribed data was conducted to make meaning of the collected data. Colours were assigned to sentences in the transcripts to indicate potential patterns. MM and MZ identified and extracted phrases and/or direct participant quotations from the data. Then codes were derived from the data set and grouped into small and meaningful chunks of data. The codes with a similar meaning were grouped to form sub-themes, which were further categorized into themes. Coauthors held several meetings to discuss and review the identified themes for a consensus. Three researchers in nursing and midwifery education with qualitative backgrounds (EC, FK & EC), who did not participate in data analysis, validated the themes to ensure that they were a true reflection of the collected data.

### Rigour

To ensure rigour, the researcher used a framework by Lincoln and Guba (1985), who recommended four criteria for promoting the trustworthiness of study findings, which include: credibility, confirmability, dependability and transferability. Credibility was achieved through a member checking strategy which was used to confirm the authenticity of the conclusions made from the participant’s explanations. Three transcripts were selected at random for member checking to confirm the authenticity of the conclusions made from the data. An audit trail which included raw data from 13 newly qualified nurse-midwives and 6 key informants, thematic categories, process notes and interpretations were kept to establish confirmability of the results. Experienced qualitative researchers were involved in data analysis to review the data analysis procedures and the themes that emerged from the data, which enhanced the dependability of the findings. Further, the research process was clearly reported to ensure the transferability of the results.

## Study findings

### Demographic characteristics of participants

Data were collected from 19 participants (13 newly qualified nurse-midwives and 6 key informants). The participants had different educational qualifications and experiences. Eight nurse-midwife technicians had a diploma in nursing and midwifery and five registered nurse-midwives had a degree in nursing and midwifery obtained within Malawi. The age of the participants ranged from 24 to 34 years. The clinical practice experience of the newly qualified nurse-midwives ranged from 6 to 24 months (see Table 1).

**Table 1:** Demographic characteristics of newly qualified nurse-midwives. *n* = 13

Variable	Frequency	Percentage
**Sex**		
Male	4	31
Female	9	69
**Age**		
21–25	5	38
26–30	7	54
31–35	1	8
**Qualification**		
Bachelor of Science in Nursing & Midwifery	5	38
College Diploma in Nursing & Midwifery	8	62
**Work experience**		
6–12 months	7	54
13–18 months	3	23
19–24 months	3	23

Three females and three male key informants within the age range of 33 to 43 years participated in this study. Two of the key informants had a master’s degree in reproductive health, while four had a bachelor’s degree in nursing and a university certificate in midwifery. Their clinical experience in managerial positions ranged from 6 to 16 years (see Table 2).

**Table 2:** Demographic characteristics of key-informants. *n* = 6

Variable	Frequency	Percentage
**Sex**		
Male	3	50
Female	3	50
**Age**		
31–35	3	50
36–40	2	33
41–45	1	17
**Qualification**		
Bachelor’s Degree	4	67
Master’s Degree	2	33
**Work experience**		
6–12 years	4	67
13–18 years	2	33

### Themes

**Fig. 1:**
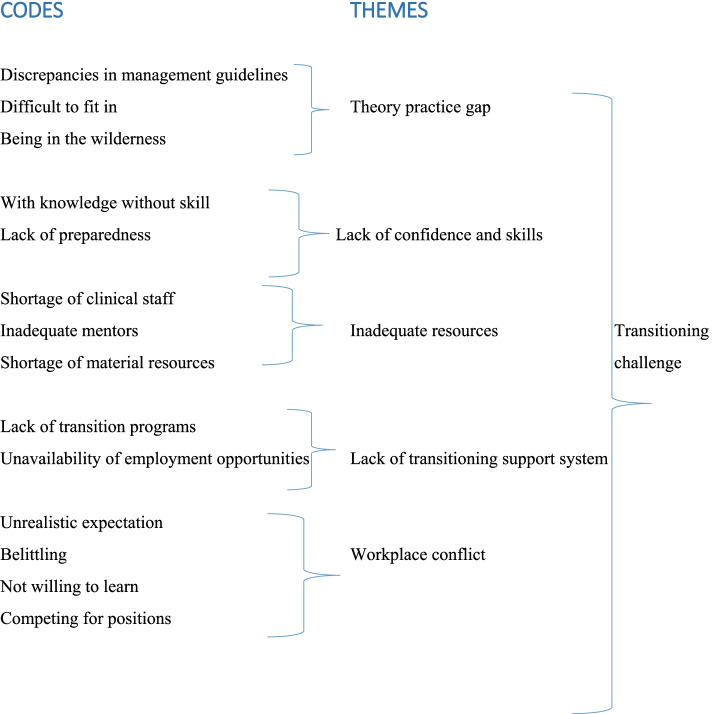
Coding tree

The study presents practical challenges that impeded the smooth transition of newly qualified nurse-midwives during the first few months of their practice at the selected hospitals. Five themes were identified and these are: (1) Theory practice gap; (2) Lack of confidence and skills; (3) Inadequate resources; (4) Lack of transition support system; and (5) The workplace conflict (see Fig. 1). These themes are presented in the sections below. The findings are supported by participant quotes, which are presented using codes to ensure participants’ anonymity.

### Theory practice gap

Regardless of education qualification on entry to practice, newly qualified nurse-midwives discussed discrepancies between content covered in class and how care was discharged in practice. While they trusted the knowledge and skills they acquired during their education, they were challenged with management guidelines for conditions in hospitals, which were different from what was covered in the classroom. This was often linked to utilising outdated industry resources (practice guidelines and standards) during their education as narrated by one participant:

“*Upon reaching the ward, I found that most of the guidelines like HIV guidelines, and some reproductive health standards had changed. There were also new things like CPAP (continuous positive airway pressure). So it was really tough for me as I was referring to old things, yet, the practice had changed on the ground” (*NMT-F- P4).

Some participants had difficulties blending in following a lack of adequate knowledge of the hospital’s management guidelines that led them to ignorantly apply theoretical knowledge from education to practice.

“*It was kind of difficult for me to fit in because when I was trying to do what I knew from training, people were always against me. They could say, we don’t follow theory rather hospital policies and management guidelines…..What is learnt in theory is not always what is on the ground. It is very different”* (RNM-F-P1)

*“I wanted to manage my clients inline with what I learnt in class, only to realize that in practice most conditions are managed differently. The situation made me feel lost in wilderness*” (NMT-F-P2)

It is evidenced by the above quotes that newly qualified nurse-midwives felt unprepared for practice in the hospitals because of the use of outdated clinical practice guidelines and standards during their education. This made them feel confused as they were criticized by experienced midwives that their knowledge and skills were below standard.

### Lack of confidence and skills

Lack of confidence and skills was a dominant theme within the study that both key informants and newly qualified nurse-midwives discussed at length. Key informants indicated that newly qualified nurse-midwives lacked the skills and confidence to care for the mothers and their newborns safely and competently despite having the knowledge. Exemplars below explicate this:

*“Theoretically they are well prepared, but most of them are not skillful….. they struggle to apply theory into practice”* (KEY-INFO-M-P5)

“*Theoretically they are equipped for practice, but they lack confidence….. If confidence is gained it is much easier for them to master the practical content while applying what they covered while training*” (KEY-INFO-M-P4).

Newly qualified nurse-midwives also attested to feeling incompetent. Lack of confidence and skills to perform procedures was unanimously discussed by the majority. See select quotes below.

“*At first, since I was just coming from college, I had more knowledge compared to practical skills. It took me time to adapt and acquire the necessary skills to match my level of theoretical knowledge acquired during training”* (RNM-M-P3).

“*I was excited when I got a job. But seriously, coming to the ward, I felt not prepared to provide care unsupervised. I even told them that I was not comfortable to do anything in nursery without support from colleagues*…..*and for the first 2 weeks, I was calling experienced staff or colleagues to be there with me whenever I was doing something. I wanted to at least be guided when doing the procedure until I gain the skills”* **(**NMT-F-P2).

From the above quotes, newly qualified nurse-midwives acknowledged the fact that they had more knowledge than skills during their initial states of practice and they relied on experienced midwives for guidance.

### Inadequate resources

Data analysis revealed that inadequate experienced nurse-midwives, mentors and material resources were major challenges that affected newly qualified nurse-midwives’ transition to practice. Participants highlighted the shortage of experienced nurse-midwives to support the new graduates. Additionally, participants complained about the lack of support by experienced nurse-midwives who were more focused on caring for mothers and babies than educating new graduates. One participant said:

“*We had few experienced-midwives in the wards and it was difficult for them to mentor us adequately to acquire the relevant knowledge and skills in practice. …..we had two senior midwives but they were mostly busy with patient care and managerial commitments. But honestly we needed to know what we were supposed to do, how things were done and the systems available. We needed somebody- a senior to mentor us”* (RNM-M-P5).

Most newly qualified nurse-midwives specified that they experienced heavy patient workload due to the shortage of staff in the wards. This increased their stress levels which made them find the profession not pleasing. A newly qualified nurse-midwife expressed how she felt overwhelmed in the ward and this is a representation of what many experienced.

*“….. human resource is a challenge….. Despite the nursery ward being one of the busy wards, there are times that you are alone on duty and you are expected to do all the activities…..if you have premature babies, you need to resuscitate them, you have to administer medication, you have to monitor vital signs….. it’s not easy. You cannot even have time to rest. But the sad thing is that seniors do not understand this” (*NMT-F-P2)

“*Sometimes I could work here alone in postnatal ward with 20 to 24 women. Honestly, I was not enjoying the work because it was too much for me”* (RNM-F-P4)

An excerpt below from a key informant affirms this.

“*Because of the shortage of staff, a new graduate can be on duty alone without an experienced midwife to consults in case they are stuck. This increases their stress level but eventually they get used ….. and they provide the care based on knowledge and skills gained from classroom” (*KEY–INFO–F-P1).

A lack of material resources was another challenge. The participants discussed how a severe shortage of resources affected their provision of ideal care. Lack of resources was said to challenge care outcomes and was stress-provoking and frustrating as highlighted below.

*“The transition process itself is stressing, shortage of resources magnifies the stress. You know, there are times when you fail to perform a procedure because of lack of resources. I remember we once lost a mother to postpartum haemorrhage. It is not that we did not know what to do to save her, but we did not have resources like normal saline…..You know psychologically and as a new graduate, you become affected and obviously you cannot enjoy the profession….. the issue of resources was a very big challenge for me”* (NMT-M-P3).

Key informants added,

***“****For the new graduates to transition well from education to practice, they need the required resources. If the resources are not there, they improvise and therefore, they do not provide the expected quality of care. (*KEY–INFO–F-P1)

“O*ne of the barriers is lack of resources. When you are at school you learn the ideal, even the clinical lab have manikins …..Those are very ideal in terms of the resources that can be there. But when you come to the practical area, resources are not quate as much”* (KEY-INFO-M-P4).

The above participants’ quotes show that the lack of human and material resources in the maternity units affected the transition of newly qualified nurse-midwives to practice. Most of them felt unsupported mainly because of increased workload, shortage of experienced staff who could mentor them and a lack of equipment to enable them to better support patients.

### Lack of transitioning support system

Another salient theme that was identified from the participant’s narratives was the transitioning support system. Both newly qualified nurse-midwives and key informants highlighted a lack of formally instituted transition support programs in the midwifery units. Newly qualified nurse-midwives indicated that they were seeking support from any midwife on duty. One participant narrated this:

“*We were not under any program. The first week I was just working with anyone who was on duty for support. Later, I could follow or consult peers and seniors whom I thought were competent in their midwifery skills* (NMT-F1-P5).

A key informant agreed to the cause and said:

*“Currently there is no formal program as for our hospital or department to assist the graduates during the transition period***” (**KEY-INFO-M-P5)

Participants expressed their perception of learning to be difficult during the transitioning process if mentorship was not readily available. One participant said:

“T*ransition is always difficult without support. It is like you are coming from a place where you were used to and you are going to a new place where you do not really know what happens there…..so in such situations mentorship is needed” (*RNM-F-P1).

Another difficult factor in transitioning was the unavailability of employment opportunities after graduation. Participants mentioned that waiting times for employment after graduating ranged from 8–13 months. This delay was pinpointed to cause a loss of confidence and skills as highlighted below.

“The *challenge we have in Malawi is the long waiting time for newly qualified nurse midwives to be employed. By the time you are employed you have forgotten all that you learnt at school. I waited for a year for employment. When I got the job, I felt like someone who has not been trained in midwifery because it was difficult to recall the management of conditions.” (*NMT-F-P2)

“*It took a long time from graduation to when I started practising as a midwife. I almost forgot everything. But support from experienced midwives helped me to get on track. By and by I started remembering the management of most conditions.”* (NMT-F- P4)*.*

In agreement, key informants shared this;

“*These graduates’ nowadays are taking longer to get employed by health facilities. So you know nursing is not just knowledge but skills too. When they stay long without practising, they start forgetting”* (KEY–INFO–F-P6).

“*Unlike previously….. there was no waiting period between graduation and employment. Currently, most graduates would have qualified maybe 3 or 4 years ago but have not been employed. So, this gap is an issue”* (KEY-INFO-M-P4).

It is evident from the above information that clinical settings had no formal programs to better support newly qualified nurse-midwives with their transition to practice experience. This affected their transition experience as they were unfamiliar with the routines in a new clinical setting. The transition experience of the graduates was also impacted by the delay in employment after graduation. Many of the graduates waited more than 8 months after graduation to be employed and this led to forgetting important clinical skills and procedures.

### Workplace conflict

In this theme, participants discussed the expectations that qualified midwives had on newly qualified nurse-midwives and how this affected their learning negatively. Experienced midwives expected newly qualified nurses to be knowledgeable and skilled, and they were spiteful in events where newly qualified nurses fell short of these expectations. In such instances, qualified midwives were not disposed to teach the newly qualified nurse-midwives. Following this, newly qualified nurse-midwives withdrew from seeking help whenever they were stuck. This is what some said:

“*Some nurses could say, you have a degree and you know all these things. Why are you asking me? Next time you can’t ask that person again for guidance and definitely you won’t learn how that skill is performed”* (RNM-F-P1).

“*I had to find some midwives who were not giving room to teach or mentor us. They could say, you have just graduated from school …..we thought that qualifies you are knowledgeable and skilled?”* (RNM-M-P5)

Some bemoaned the actions of some midwives who belittled them. Belittling affected their confidence and morale. One participant said:

“You *come at a place and they [experienced midwives] treat you like you don’t know anything. That affects someone as a result you underrate yourself. I thought I could not perform or do anything because of the way they handled me…..they could not let me make decisions for the care I could provide to my clients”* **(**NMT-F-P2).

Some key informants highlighted that some newly qualified nurse-midwives who graduated with a bachelor’s degree struggled during the transition period because they were not willing to learn from qualified midwives. They considered themselves to be equally knowledgeable and at par with the ward in-charges or other experienced midwives.

*“Some do come with the mentality that they know it all because they have attained a degree. They feel they have all the knowledge and skill. Nobody can tell them what to do…..This affects their relationship with experienced staff as none expresses interest to support them. As a result they do not learn the needed skills and their transition becomes difficult and stressful”* (KEY–INFO–F- P3)

It was also noted that some nurse-midwives with a bachelor’s degree were competing for positions mainly those that were allocated in the same ward. One key informant narrated:

*“Many registered nurse-midwives do fight for positions. When two or more have been allocated in the same ward, they start competing for positions. They want to be ward in-charges. This affects their focus and efficiency in discharging their duties as they spend time fighting for the position*” **(**KEY-INFO-M-P5).

From the above participants’ quotes, it is evident that some experienced qualified midwives had no interest to support the new graduates because they expected them to have the required skills and knowledge. On the other hand, the behaviours of some graduates also influenced their transition experience as some of them were not willing to learn from experienced staff with lower qualifications than them.

## Discussion

The study findings revealed that newly qualified nurse-midwives encountered several challenges in the midwifery units where they were practising. From our findings, the transition of the newly qualified nurse-midwives was affected by the discrepancies between the management guidelines taught in class and how midwifery conditions were being managed in practice, which was frustrating and provoked anxiety. These findings are not unique to Malawi alone. The theory–practice gap was also found in a Canadian study where new midwives experienced feelings of frustration as they found differences in philosophies of care [32]. Similarly, stress and anxiety were reported in an Australian study following the inability to provide the expected midwifery care due to differences in the content taught in class and the delivery of midwifery care in the healthcare setting [9]. While health institutions in other countries have varied policies and management guidelines for conditions, in Malawi, management guidelines are devised and distributed by the ministry of health. There is, therefore, a need to strengthen the collaborative partnership between education institutions and the midwifery industry in Malawi to be able to share up-to-date resources for teaching nursing and midwifery students. In addition, the midwifery regulatory authority and education institutions and the midwifery industry need to to work together revise the pre-registration midwifery curriculum to ensure that the theory taught in the classroom matches with the current clinical midwifery practice.

Our findings reveal that newly qualified nurse-midwives feel incompetent when performing skills as attributed to lack of experience which aligns with findings of studies done in Australia, New Zealand and Canada [3, 5, 10]. In these studies, it was established that new graduate midwives are not ready and confident to practice the full scope of midwifery practise upon completion of their education, they needed support from experienced midwives [3, 5, 10]. Authors from Swaziland have associated a lack of clinical skills in newly graduated nurses with poor pre-service preparation [33]. These authors established the limitation of the education system to prepare students for practice and the failure of hospitals to adequately support and induct graduates into the system as reasons for poor performance. Findings from three studies conducted in Malawi resonate with findings from a study conducted in Swaziland where poor performance was attributed to a lack of emphasis in clinical teaching and supervision by nursing colleges, poor support from clinical staff and lack of skills among clinical staff [34–36]. There is a need for the Malawian Government to work with the midwives’ regulatory body to establish effective transition support programs in hospitals. This could be possible if the national frameworks/guidelines for practice could revised to provide transition strategies for the newly qualified midwives such as formal mentorship and comprehensive orientation that could better support them to adjust to their new roles for competency and confidence.

The study further identified a shortage of resources including experienced midwives to mentor newly qualified nurse-midwives in practice. Support from experienced nurses is important as highlighted in Australian and Canadian studies where new graduate midwives considered support and mentorship from experienced midwives as a “life raft” during the transition period as it improved confidence and a sense of safety [9, 32]. Another finding in this study that affected proper support for the newly qualified nurse-midwives was heavy workloads. The heavy workload increased the stress levels of newly qualified nurse-midwives, which is similar to the findings from other studies [4, 18, 33]. Shortage of staff and heavy workload have an implication on midwifery leaders’ ability need to increase the workforce in Malawian hospitals considering that it is one of the contributing factors to the provision of low quality patient care, burnout and high staff turnovers [37–40]. Although in this study graduates had no intention of leaving the profession, feelings of increased stress and decreased morale were high, which may lead to midwives’ attrition in the three hospitals. While the use of work and workload frameworks in some countries has been shown to standardise workloads to improve fairness among midwives, this is not the case with Malawi. In Malawi, midwife-patient ratio is not formally defined, as such midwives work according to the demands of the day. We recommend the adoption of such frameworks to avoid the burnout that often results from the heavy workload.

Our study found that it was taking about 8–13 months for the newly qualified nurse-midwives to be employed after graduation leading to a loss of confidence and skill. This is not unique to Malawi alone, findings from other African and Asian countries show that many newly graduated health professionals including midwives face problems finding employment soon after graduation [41, 42]. Hughes (2011) indicates that the long delay between qualification and employment is stressful. Midwifery is one of the professions that rely on hands-on practice for the perfection of skills and the development of confidence [5]. When a midwife is disengaged from practice for some time, they lose important clinical skills to enable them to perform effectively when they get employment. This has implications for the Malawian government and its development partners need to create policies or develop strategies like an internship that could help to reduce the period that the graduates wait to get employed. This could help keep graduates in the system and prevent the loss of important clinical skills.

Unsupportive attitudes of the experienced midwives were also identified as a barrier to smooth transitioning by newly qualified midwives. The newly qualified nurse-midwives with degrees in our study reported that they were considered highly qualified and knowledgeable and therefore, did not require support from experienced midwives. Similarly, Tembo et al. (2019) recruited newly qualified registered midwives who complained of negative attitudes by older midwives. Literature shows that poor professional relationships impact new midwives’ confidence and clinical performance negatively [4]. However, support from midwives in a clinical setting has proved to create a conducive environment where newly qualified nurse-midwives feel secure and confident and their clinical performance is enhanced [4, 16].

Furthermore, in this study, we found that most newly qualified nurse-midwives with degrees wanted to be ward in-charges. This created tension when two or more of the registered nurse-midwives were allocated to the same ward. This behaviour has a negative impact on graduates’ integration into the workforce, as at this point they are novices and need support to smoothly transition into practice. The implication is that it affects the relationship between the graduates and the experienced midwives leading to decreased morale and a negative perception of the transition period by the graduates [43]. Freeling and Parker (2015) highlight that the self-imposed in-charges may face resistance from other graduates leading to increased levels of anxiety and a feeling that they are not accepted in the midwifery setting [44]. Many studies conducted on the transition to practice for nurses and midwives have recommended the use of formally instituted transition support programs. However, many of these have been implemented in high-income countries with very few in Africa. These programs have not been piloted in Malawi. Randomised control trials are, therefore, warranted, to test and determine the effectiveness of transition support programs for Malawian new graduate nurse-midwives.

### Strengths and limitations of the study

Data were collected from registered nurse-midwives with a degree in nursing and midwifery, nurse-midwives technicians with diplomas and key informants. This helped to enrich the study findings as the ideas presented were from a wide perspective, which helped to reduce bias in the study and improve credibility of the findings. In addition, the study has provided valuable insight into the challenges newly qualified nurse-midwives encounter in midwifery settings from the perspective of Malawian nurse-midwives. Notwithstanding the strengths, the study has a number of limitations. First and foremost, this study was conducted in three selected midwifery settings as such the findings may not represent the ideas of other nurse-midwives in other settings. However, the findings of this study will be made available for readers to decide on generalizability to a similar context based on the research process and the available study findings. Secondly, this is a qualitative study, as such, subjectivity in the analysis and interpretation of the data may not be ruled out. However, the process of data analysis involved a group of researchers with expertise in qualitative research who examined the transcripts and the coding process and the themes to validate the study findings. In addition, we also asked the participants to review the transcripts after data collection to validate and improve the credibility of the findings.

## Conclusion

The study findings, contribute to a better understanding of the transition period of newly qualified nurse-midwives and the challenges they face during the transition period in midwifery settings. For most newly qualified nurse-midwives in Malawi, the transition from education to practice has many challenges. Delays in recruitment, changes in management guidelines, lack of properly instituted transition strategies, shortage of experienced midwives, workload, workplace conflict and lack of confidence by newly qualified nurse-midwives are the challenges that hindered the smooth transition from education to practice. We acknowledge the importance of orientation guidelines and transition strategies, which if developed and implemented, could improve the transition process with better outcomes. A collaborative partnership between colleges and hospitals is crucial in finding solutions and change outcomes for the newly graduated midwives for a better transition to practice.

## Supplementary Information

**Additional file 1:** **Appendix 1.** Interview guide for newly qualified nurse-midwives. **Appendix 2.** Interview guide for key informants

## Data Availability

Data is available with the corresponding author and may be made available upon request.
