# Supplementary material for: Challenges experienced by newly qualified nurse-midwives transitioning to practice in selected midwifery settings in northern Malawi
Source: BMC Nurs. 2022 Aug 25;21:236. doi: 10.1186/s12912-022-01012-y (PMC9413903; doi:10.1186/s12912-022-01012-y)
Supplement: Supplementary file 1 — Additional file 1: Appendix 1. Interview guide for newly qualified nurse-midwives. Appendix 2. Interview guide for key informants [file 12912_2022_1012_MOESM1_ESM.docx]

**Appendix 1:**  **Interview Guide for Newly Qualified Nurse-midwives**

INTERVIEW QUESTIONS

1. What was your experience like in the first few months as a new newly qualified nurse-midwife?
   1. What were the challenges?
   2. What were the successes?
2. Describe the orientation process you experienced when you started as a newly qualified nurse-midwife?
   1. Who oriented you?
   2. How long was the orientation?
   3. Were you under any program (internship, mentorship, preceptorship or any other)
3. How prepared were you to take up the role of qualified midwife?
   1. What does being well prepare mean to you?
   2. Do you feel like you have the confidence and skills needed to take up the new role?
4. What type of support is provided to you in the ward?
   1. Elaborate more
5. What type of support structures are in place that supports you?
   1. Describe each structure
   2. Which one do you find helpful? And why?
6. What factors facilitated/influenced your transition from student to professional role?
7. What factors impeded/affected your transition process?
8. Have you ever considered leaving the midwifery profession?
   1. If yes, Why?
   2. If no, Why?
9. Describe factors that can assist to have a successful transition from student to qualified midwife?

**Appendix 2: Interview Guide for Key Informants**

INTERVIEW QUESTIONS

1. How long have you worked in maternity?
2. Describe the orientation process you provide to newly qualified nurse-midwives?
3. What is your opinion on newly qualified nurse-midwives preparedness to take up the role of a qualified midwife?
4. Describe strategies you have in place to support newly qualified nurse-midwives transitioning from training to practice?
5. Which strategies can you recommend and why?
6. What factors facilitate newly qualified nurse-midwives transition?
7. What do you think are the barriers to successful transition?
8. What factors if available would have helped promote the transition process?
9. What is your general comment in regards to newly qualified nurse-midwives transition to practice?
